# Hyperfocus symptom and internet addiction in individuals with attention-deficit/hyperactivity disorder trait

**DOI:** 10.3389/fpsyt.2023.1127777

**Published:** 2023-03-16

**Authors:** Sayuri Ishii, Shunsuke Takagi, Nanase Kobayashi, Daisuke Jitoku, Genichi Sugihara, Hidehiko Takahashi

**Affiliations:** ^1^Department of Psychiatry and Behavioral Sciences, Tokyo Medical and Dental University Graduate School, Tokyo, Japan; ^2^Sleep Research Institute, Waseda University, Tokyo, Japan; ^3^Center for Brain Integration Research, Tokyo Medical and Dental University, Tokyo, Japan

**Keywords:** ADHD, hyperfocus, internet addiction, mediation analysis, web-based study, questionnaire study

## Abstract

**Background:**

Hyperfocus symptom is the intense concentration on a certain object. It is a common but often overlooked symptom in those with attention-deficit/hyperactivity disorder (ADHD). Hyperfocus disrupts attention control and results in a focus on inappropriate behaviors. It allows individuals to focus on internet use and make them use internet excessively. This excessive internet use can lead to an addiction. This study investigated the status of IA and hyperfocus, the mediation effect of hyperfocus in relation to IA, and the relationship between ADHD subtypes and hyperfocus in those with ADHD symptoms.

**Methods:**

This web-based cross-sectional study included 3,500 Japanese adults who completed internet-based questionnaires, which included the Adult ADHD Self-Report Scale (ASRS), Internet Addiction Test (IAT), and Hyperfocus Scale (HFS) to assess ADHD symptoms, internet dependence, and hyperfocus symptoms, respectively. The mediating role of HFS in the relationship between ASRS and IAT was assessed by mediation analysis. To analyze the relationship between hyperfocus symptoms and ADHD subtypes, we compared the correlation of HFS with the Inattention and Hyperactive Scores of ASRS.

**Results:**

ADHD traits were associated with higher IAT scores (*p* < 0.001) and higher HFS scores (*p* < 0.001). Mediation analysis and bootstrap testing showed that HFS significantly mediated the association between ASRS and IAT. Analyses of ADHD subtypes demonstrated that HFS was significantly correlated with the Inattention (*R* = 0.597, *p* < 0.001) and Hyperactive (*R* = 0.523, *p* < 0.001) Scores. The correlation between HFS and the Inattention Score was significantly higher than that between HFS and the Hyperactive Score (*p* < 0.001).

**Conclusion:**

Our findings suggest that hyperfocus may play an important role in addictive behavior in ADHD as a manifestation of attentional control malfunction.

## 1. Introduction

Attention-deficit/hyperactivity disorder (ADHD) is a chronic lifelong condition that is mainly characterized by symptoms such as inattention, hyperactivity, and impulsivity (1). ADHD is one of the most common neuropsychiatric disorders in children and adolescents, with an estimated prevalence of about 4–12% worldwide (2–4). Furthermore, more than half of individuals with ADHD continue to have symptoms into their adulthood (5). The prevalence of ADHD in the adult population is estimated to be as high as 3–4% (6–8). The main symptoms of ADHD make it difficult for people to sustain their attention on certain activities and tasks. In addition to the main symptoms, some individuals with ADHD have other symptoms and comorbidities (1, 9). As the other symptoms, disorganization prioritizing, poor time management skills and frequent mood swings are frequently pointed out (1, 3). Research indicates that ADHD is often associated with comorbid psychiatric disorders such as depression, bipolar disorders, anxiety disorders (10–12), and substance use disorders (13).

Among the comorbid conditions of ADHD, “hyperfocus” symptom, an experience of very intense concentration or “overconcentration”, has been recognized by clinical practitioners (14). This condition is described as “a state of being laser-focused (concentrated) on a particular event or topic” (15). Although there is no established definition of hyperfocus, prior research has revealed several common characteristics in hyperfocus (which suggest the definition) as follows: (a) hyperfocus is characterized by an intense state of concentration/focus; (b) in the hyperfocused state, unrelated external stimuli do not appear to be consciously perceived (sometimes reported as a diminished perception of the environment); (c) to engage in hyperfocus, the task has to be fun or interesting to the individual; and (d) in the hyperfocused state, task performance improves (16). Based on the definition, a questionnaire scale to measure the tendency to hyperfocus has been published and validated (17). A study showed that 78% of people with ADHD experienced hyperfocus symptoms at least once in their lives (15). Hyperfocus can be regarded as a manifestation of excessive attentional dysfunction related to ADHD. While hyperfocus make individuals with ADHD sustain attention on a specific task, it can also lead to difficulties in managing time and prioritizing tasks, causing further problems in daily life.

Although hyperfocus may sometimes improve abilities in ADHD individuals, such as productivity (18), it can cause problems. Physical problems in ADHD such as headache may be attributable to the hyperfocus state (19). Hyperfocus can also cause social problems. For instance, unrestricted concentration on unproductive tasks due to hyperfocus can lead to decreased academic and work productivity. Hyperfocus can undermine good relationships with other individuals, which may lead to social isolation and low social functioning. Whether people with ADHD experiences hyperfocus or inattention can be context-specific, as different tasks and environments may trigger different patterns of attention and behavior. For example, someone with ADHD may have difficulty paying attention in a lecture or meeting they are not interested in, but may become highly focused when working on a task they are interested in.

These features, from concentration on inadequate behavior to social dysfunction, are similar to those in people with behavioral addiction (20), which is characterized by an uncontrollable impulse to engage in a certain behavior despite the harmful consequences. For instance, internet addiction (IA), a type of behavioral addiction, is one of the most serious comorbidities of ADHD (21). A study showed that 56% of people with ADHD also had IA (22). IA has serious impacts on the quality of life, ranging from loss of physical strength and bone density due to reduced physical activity (23), sleep disturbance due to the inversion of day and night, increased aggression, lethargy (24), and depression (25) to social problems such as poor grades, truancy, and poverty (26).

Despite the findings on hyperfocus symptom and IA in ADHD, there are still no reports, to the best of our knowledge, on the relationship between hyperfocus symptoms and IA in people with ADHD. In this study, we conducted a web-based questionnaire survey of a large sample of Japanese adults (*N* = 3,500) to investigate the (1) status of IA and hyperfocus in people with ADHD symptoms, (2) mediation effect of hyperfocus in relation to IA in people with ADHD symptoms, and (3) relationship between the subtype of ADHD and hyperfocus.

## 2. Methods

### 2.1. Participants

The study protocol was approved by the Ethics Committee of Tokyo Medical and Dental university, Tokyo, Japan. This was a web-based, cross-sectional study that recruited the participants *via* Rakuten Research Inc., an online marketing research company that holds ~2.2 million Japanese enrollments. The survey was conducted in February 2021. An email containing a link to an online questionnaire was randomly sent by the research company to individuals throughout Japan who were stratified by district, gender, and age. The participants' ages ranged from 15 to 79 years. An informed consent form was provided to participants *via* the survey website. We received 3,500 responses, which were used in the analyses.

### 2.2. Assessments

The questionnaires consisted of demographic questions, the Adult ADHD Self-Report Scale version 1.1 (ASRS) to evaluate ADHD symptoms, the Internet Addiction Test (IAT) to measure the degree of dependence on the Internet, and the Hyperfocus Scale (HFS) to estimate the tendency to hyperfocus.

#### 2.2.1. Demographic questionnaire

Participants provided answers related to their gender, age, and birthplace.

#### 2.2.2. Adult ADHD self-report scale version 1.1

In this study, we used the ASRS to screen for possible ADHD and determine the severity of the ADHD trait. ASRS was developed by the World Health Organization and has been widely used in epidemiological studies on ADHD (27–29). The six-item ASRS was designed as a tool to screen for ADHD in adults (aged ≥ 18 years) based on the diagnostic criteria of the Diagnostic and Statistical Manual of Mental Disorders, 4th edition, Text Revision (30). This scale is standardized and well-validated for the assessment of current ADHD symptoms. Each item requires participants to indicate how often a particular symptom has occurred over the past 6 months on a five-point Likert scale from 0 (never) to 4 (very often). The total score is summed and can range from 0 to 24. Based on the classification criteria recommended by Kessler et al. (28), participants were classified as “possible ADHD” if their ASRS score was ≥14, and as “non-ADHD” if the score was < 14. The ASRS score was used as a continuous variable in our mediation analyses, with the sum of the first four items of ASRS relating to inattention symptoms as the Inattention Score and the sum of the last two items relating to hyperactivity symptoms as the Hyperactive Score (31).

#### 2.2.3. IAT

IAT is a questionnaire based on the Internet Addiction Diagnostic Questionnaire, which is a list of eight addiction criteria to diagnose problematic internet use developed by Young (32). IAT consists of 20 questions that measure the presence and severity of IA and the symptoms of IA. In this context, IA is defined as compulsive online behavior that interferes with normal social interaction and increases daily stress, loneliness, anxiety, and depression. The test measures the degree of involvement in online activities using a five-point Likert scale response and classifies addictive behaviors into three categories: a total score of < 40 as “no addiction”, 40–69 as “mild signs of addiction”, and ≥70 as “severe addictive behaviors”. Studies have confirmed that the IAT is a reliable instrument that covers the main characteristics of pathological internet use with good reliability in various countries and populations (33).

#### 2.2.4. Hyperfocus scale

HFS is a questionnaire to measure the tendency to hyperfocus (14, 17). It consists of 11 items regarding the most common complaints about hyperfocus, such as “Due to excessive focusing on a work, I often neglect myself and those around me.” Participants respond on a four-point Likert scale ranging from 1 “totally disagree” to 4 “totally agree”. The total score ranges from 11 to 44, with a higher total score indicating a stronger tendency to hyperfocus.

### 2.3. Statistical analyses

First, demographic variables and scores on the ASRS, IAT, and HFS scales were compared between the possible ADHD and non-ADHD groups using unpaired *t*-tests for continuous variables and chi-square tests for categorical variables. Regarding the normality of the continuous data, if the sample size is large enough, the distribution of differences estimated by the central limit theorem approaches a normal distribution even if the population is not normally distributed. Therefore, it was determined that there would be no problem to perform the parametric test (34). Next, a mediation analysis was conducted to evaluate the indirect effect of HFS on the relationship between ASRS and IAT. According to a study by Baron and Kenny (35), the mediation model shows that the causal effect of variable independent variable can be divided into an indirect effect on dependent variable through a mediator and a direct effect on dependent variable.

HFS score is a severity scale without a cutoff point for diagnosis. ASRS and IAT are also recognized as severity scales (36), but with cutoff points for diagnosis. Therefore, all questionnaire scores were used as continuous variables in this mediation analysis. In the analysis, a bootstrap sampling procedure with 4,000 iterations was used to determine the significance of indirect effects. In the bootstrap method, the number of iterations is determined according to previous study (37). To add enough accuracy for the analysis relatively high iterations number was chosen. We considered bootstrap values with 95% confidence intervals that did not contain zero between their lower and upper limits to be significant mediators.

The effect size was determined using Cohen's d, ϕ, and Cramer's V. In general, Cohen's d values of ≤ 0.2, ~0.5, and ≥0.8 indicated small, medium, and large effect sizes, respectively. Cramer's V and ϕ values of ≥0.1, ~ 0.3, and ≤ 0.5 indicated small, medium, and large effect sizes, respectively. In this study, the effect size was confirmed by Cohen's d in the *t*-test and ϕ in the chi-square test.

Pearson's correlation coefficient was calculated between HFS and the Inattention Score and between HFS and the Hyperactive Score. After confirming that each correlation coefficient was significant, a test of significance was conducted on the difference between the two correlation coefficients according to Hittner et al. (38).

All analyses except for the correlation comparisons were performed using SPSS version 20 (IBM Corp., Armonk, NY). The correlation comparisons were performed using R (39), with the “cocor” package (40). The correlation comparisons were performed using R (36), because an R package “cocor” (37) was available to conduct this analysis. *P*-values of < 0.05 were considered statistically significant.

## 3. Results

### 3.1. Demographic data

Table 1 shows the descriptive statistics for the total sample and the groups stratified by the presence/absence of possible ADHD. The number of individuals with possible ADHD was 190 (5.4%) and that of non-ADHD individuals was 3,310 (94.6%); these values were comparable with the prevalence of ADHD in the Japanese general population (41). There were significant between-group differences with respect to gender, age, ASRS scores, IAT scores, and HFS scores.

**Table 1:** Descriptive statistics for the entire sample and for the presence/absence of possible ADHD.

**Characteristic**	**Total (*N* = 3,500)**	**Possible ADHD (*N* = 190)**	**Non-ADHD (*N* = 3310)**	***P*-value**	**Effect size**
Gender (%male)	56.5	52.6	56.7	< 0.001	0.02
Age (years) mean (SD)	48.1 (13.2)	39.4 (11.9)	48.6 (13.1)	< 0.001	0.70
ASRS mean (SD)	7.4 (4.1)	16.1 (2.6)	6.9 (3.5)	< 0.001	2.63
IAT mean (SD)	38.1 (14.8)	60.1 (18.8)	36.9 (13.5)	< 0.001	1.68
HFS mean (SD)	21.2 (5.8)	28.3 (6.0)	20.8 (5.5)	< 0.001	1.36

The unpaired t-test was used to compare continuous variables between the two groups. Pearson's chi-square test was used to compare categorical variables between the two groups.

ADHD, attention-deficit/hyperactivity disorder; ASRS, Adult ADHD Self-Report Scale; IAT, Internet Addiction Test; HFS, Hyperfocus Scale; SD, standard deviation.

The average IAT score was significantly higher in the possible ADHD group than in the non-ADHD group. In addition, the percentage of those with ≥40 points, indicating possible IA, was 85.3% in the possible ADHD group and 34.3% in the non-ADHD group. Furthermore, the percentage of those with a score of ≥70, indicating severe addictive behaviors, was 28.4% in the possible ADHD group and 1.9% in the non-ADHD group.

### 3.2. Mediation analysis

**Figure 1:**
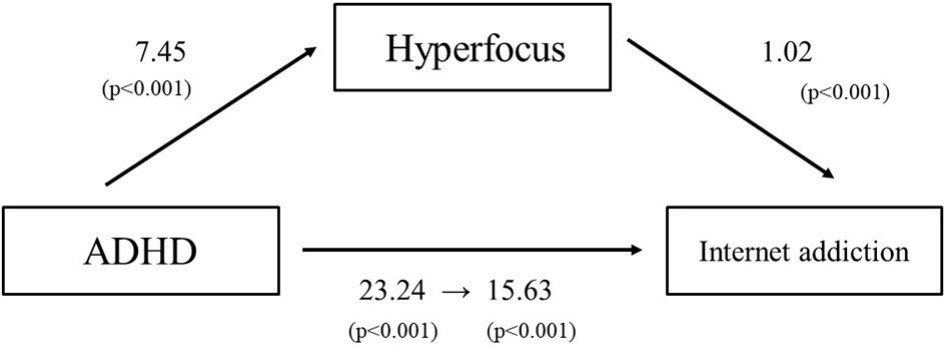
A mediation analysis was used to examine whether hyperfocus mediates the relationship between ADHD and IA tendencies. To examine the relationship between ADHD tendency and IA (c), regression analysis was performed and a positive correlation was found (*c* = 23.24, *p* < 0.001). Regression analysis was then conducted to examine the relationship between ADHD tendency and hyperfocus intensity (a), and there was a positive correlation (*a* = 7.45, *p* < 0.001). Finally, multiple regression analysis was performed with ADHD tendency and hyperfocus intensity as independent variables and IA tendency as the dependent variable, and a positive association was found. Regressing IA tendency in relation to hyperfocus tendency and testing path b revealed a significant positive association between both variables (*c*′ = 15.633, *p* < 0.001, *b* = 1.02, *p* < 0.001). The association between ADHD tendency and IA tendency was significantly mediated by hyperfocus intensity.

The model tested in this study and its results are shown in Figure 1. The regression analysis showed a significant relationship between the ASRS and IAT scores (regression coefficient = 23.24, *p* < 0.001). The regression analysis also indicated a significant relationship between the ASRS and HFS scores (regression coefficient = 7.45, *p* < 0.001). Furthermore, we added the mediating variable HFS to the equation and performed regression to examine whether HFS mediates the relationship between ASRS and IAT. In this multiple regression analysis, the path between Hyperfocus and internet addiction, examined by regressing the IAT trend on HFS, indicated a significant positive association between these two variables (regression coefficient between ADHD and internet addiction = 15.633, *p* < 0.001, regression coefficient between Hyperfocus and internet addiction = 1.02, *p* < 0.001). This indicated that the association between ASRS and IAT was significantly mediated by HFS. The direct effect of the regression analysis, with HFS as a mediating variable, remained significant, indicating that this was a partially mediated model. A bootstrap test showed that the indirect effect was significant. These results indicated that the relationship between ASRS and IAT was partially mediated by HFS.

The regression analysis and mediation analysis were also applied to the Inattention Score and the Hyperactive Score as the ASRS to IAT and HFS. Both the Inattention Score and the Hyperactive Score were shown to have significant relationship with IAT scores (regression coefficient = 2.83, *p* < 0.001; 4.40, *p* < 0.001, respectively) and with HFS scores (regression coefficient = 1.23, *p* < 0.001; 0.715, *p* < 0.001, respectively). The mediating effect of HFS to these relationships were also shown significant (regression coefficient to IAT = 2.10, *p* < 0.001; 3.14, *p* < 0.001, respectively: regression coefficient between HFS and IAT = 0.596, *p* < 0.001, 1.76, *p* < 0.001).

### 3.3. Hyperfocus in ADHD symptom profile

Pearson correlation showed significant linear relationships between HFS and the Inattention Score (*r* = 0.597, *p* < 0.001) and between HFS and the Hyperactive Score (*r* = 0.523, *p* < 0.001), with the relationship between HFS and Inattention Score showing a significantly higher correlation coefficient (*z* = 6.34, *p* < 0.001), suggesting that the hyperfocus symptom is more highly correlated with the inattention symptom than with the hyperactive symptom.

## 4. Discussion

The present study showed that the (1) ADHD group was significantly more likely to have IA and hyperfocus tendencies, consistent with previous studies (14, 16), (2) hyperfocus symptom partially mediated the relationship between IA and ADHD, and (3) hyperfocus symptom was more correlated with inattention than with the hyperactivity. To our knowledge, this study is the first study to examine the relationship between hyperfocus and IA in people with ADHD traits using a large sample. This study is also the first to examine the relationship of hyperfocus symptom in subtypes of ADHD.

This study included a large sample through an internet survey. Generally, the prevalence of possible ADHD in this study (5.4%) did not deviate from the ADHD prevalence in previous studies (42). In addition, the prevalence of IA among all participants was 37.1%, comparable to values in prior studies (43, 44). Our samples with an average age of 48.1 years seemed relatively old for a survey regarding ADHD, given that problems related to ADHD start in childhood (1) and that they are attenuated as individuals grow. However, the fact that the prevalence of ADHD and IA in this cohort did not differ from that of the general population suggests that our samples are representative with respect to ADHD characteristics and IA.

Our findings suggest that hyperfocus plays a significant role in the mechanism of progression from ADHD to IA. This study confirmed a high incidence of IA among people with ADHD as previously reported (42–44). A study reported that among the main symptoms of ADHD, inattention symptom has the most prominent influence on the tendency for IA (47). In addition, inattentive symptoms have been shown to be related to the state of dependence (46). Despite these reports, inattention symptom and addictive behavior do not appear to be directly connected, because inattention generally interferes with concentrating on certain things. Thus, one would expect reduced concentration due to inattention to lead to attenuated addictive behavior. In other words, inattention can decrease engagement with the addictive object, thus lowering the risk of dependence (47). Despite these divergent views, the results of the present study suggest that the former explanation is plausible. Hyperfocus allows individuals to become deeply engrossed in online activities. Intense focus can make it difficult for people to leave from their screens and engage in other activities, leading to increased amounts of time spent online. This excessive internet use can lead to an addiction. Addition to it, Hyperfocus might play a role in developing internet addiction by activating the brain's reward center and triggering the release of dopamine (48). The release of dopamine reinforces the behavior and creates a feedback loop, making it more likely that individuals will continue to engage in the behavior. People with ADHD seeks immediate rewards. Hyperfocus could be the reward by this effect on the reward center.

Our findings suggest that hyperfocus is associated with IA in individuals with ADHD, while inattention has been shown to be related to IA (45, 46). Hyperfocus and inattention can be considered to be results of attention control malfunction in ADHD (49, 50). Inattention can be regarded as deficient attentional dysfunction, and hyperfocus can be regarded as excessive attentional dysfunction (51). In this context, people with ADHD might have inattention and hyperfocus simultaneously with variety of severity, and they tend to have IA mainly due to their hyperfocus symptom.

There are some limitations to this study. First, possible ADHD was determined by the cutoff score of the ASRS without structured clinical interviews. Second, ASRS was used for people aged < 18 years in this study, although the questionnaire is designed for people aged ≥18 years. Third, because of the nature of an internet survey, the response rate could not be calculated (52). Fourth, the detailed mechanism of hyperfocus in the relationships between ADHD and IA is still unclear. Therefore, further research to prove hyperfocus can act as a mediator are needed.

The present study revealed that hyperfocus, along with ADHD tendencies, was significantly associated with IA. Although hyperfocus is often overlooked as one of the symptoms of ADHD, it may play an important role in the development of IA. Furthermore, hyperfocus symptoms were more common in inattentive types of ADHD as a manifestation of malfunctioning attentional control. Our findings shed light on the relationships between hyperfocus symptoms and addictive behaviors in ADHD.

## Data availability statement

The raw data supporting the conclusions of this article will be made available by the authors, without undue reservation.

## Ethics statement

The studies involving human participants were reviewed and approved by Ethical Committee of Tokyo Medical and Dental University. Written informed consent for participation was not required for this study in accordance with the national legislation and the institutional requirements.

## Author contributions

All authors made a significant contribution to the work reported, whether that is in the conception, study design, execution, acquisition of data, analysis and interpretation, or in all these areas, took part in drafting, revising or critically reviewing the article, gave final approval of the version to be published, have agreed on the journal to which the article has been submitted, and agree to be accountable for all aspects of the work.
